# TDAG8 involved in initiating inflammatory hyperalgesia and establishing hyperalgesic priming in mice

**DOI:** 10.1038/srep41415

**Published:** 2017-02-01

**Authors:** Shih-Ping Dai, Ya-Han Huang, Chung-Jen Chang, Yu-Fen Huang, Wei-Shan Hsieh, Yasuhiko Tabata, Satoshii Ishii, Wei-Hsin Sun

**Affiliations:** 1 Department of Life Sciences, National Central University, Jhongli, Taoyuan City, Taiwan; 2 Department of Biomaterials, Institute for Frontier Medical Science, Kyoto University, Kyoto, Japan; 3 Department of Immunology, Graduate School of Medicine, Akita University, Akita city, Japan.

## Abstract

Chronic pain, resulting from injury, arthritis, and cancer, is often accompanied by inflammation. High concentrations of protons found in inflamed tissues results in tissue acidosis, a major cause of pain and hyperalgesia. Acidosis signals may mediate a transition from acute to chronic hyperalgesia (hyperalgesic priming) via proton-sensing G-protein-coupled receptors (GPCRs). The expression of T-cell death-associated gene 8 (TDAG8), a proton-sensing GPCR, is increased during inflammatory hyperalgesia. Attenuating TDAG8 expression in the spinal cord inhibits bone cancer pain, but whether TDAG8 is involved in inflammatory hyperalgesia or hyperalgesic priming remains unclear. In this study, we used TDAG8-knockout or -knockdown to explore the role of TDAG8 in pain. Suppressed TDAG8 expression delayed the onset of inflammatory hyperalgesia and shortened hyperalgesic time in mice. In a dual acid-injection model (acid [pH 5.0] injected twice, 5 days apart), shRNA inhibition of TDAG8 shortened the duration of the second hyperalgesia. Similar results were found in TDAG8-deficient mice. The dual administration of TDAG8 agonist also confirmed that TDAG8 is involved in hyperalgsic priming. Accordingly, TDAG8 may mediate acidosis signals to initiate inflammatory hyperalgesia and establish hyperalgesic priming.

Chronic pain resulting from injury, infection, arthritis or cancer often accompanies inflammation, which heightens the pain experience by increasing the sensitivity of nociceptors to both thermal and mechanical stimuli^1^. The inflammatory “soup” contains relatively high local proton concentrations that causes tissue acidosis and contributes directly to pain and hyperalgesia^2^. Acidosis was recently suggested to mediate a cellular kinase-signalling switch (establishing a priming state) that regulates the transition from acute to chronic pain^3^,^4^,^5^.

Proton-sensing receptors play important roles in the transduction of acidosis signals. Acid-sensing ion channel 3 and transient receptor potential/vanilloid receptor subtype 1 (TRPV1) participate in establishing acid-induced priming in muscle nociceptors^6^. In cutaneous nociceptors, a signal switch from protein kinase A (PKA) to protein kinase C ε (PKCε) is involved in acid- or complete Freund’s adjuvant (CFA)-induced priming^3^, but which receptors are upstream of kinase signalling to establish the hyperalgesic priming remain unclear.

T-cell death-associated gene 8 (TDAG8), a member of the OGR1 family, was identified as a proton-sensing G-protein–coupled receptor with full activation at pH 6.4 to 6.8. Activation of TDAG8 leads to activation of the cAMP-PKA pathway, increased intracellular calcium level and Rho activation^7^,^8^,^9^. About three quarters of small-diameter dorsal root ganglia (DRG) neurons and about half of isolectin B(4) (IB_4_)-positive non-peptidergic neurons express TDAG8^10^. TDAG8 expression is increased in CFA-induced inflammatory pain, and its activation by protons sensitizes TRPV1 function, especially in IB_4_-positive neurons^11^. Inhibiting spinal PKA activation mediated by TDAG8 reduced bone cancer pain^12^. As well, CFA-increased TDAG8 expression was reduced after PKC inhibitor treatment, which suggests that TDAG8 plays a role in maintaining the hyperalgesia^3^. Moreover, TDAG8 mediates acid-inhibited cytokine production in macrophages, T cells, and microglia^13^,^14^,^15^, so TDAG8 may be a negative factor regulating inflammation.

In this study, we used TDAG8 shRNA to knock down TDAG8 gene expression for determining whether TDAG8 is involved in the transition from acute to chronic pain. Knocked-down TDAG8 expression delayed the initiation of mechanical hyperalgesia in mice with acid, carrageenan, or CFA injection. Inhibition of TDAG8 expression also reduced cAMP accumulation and Ca^2+^ signals in DRG neurons. With dual acid injection, knocked-down TDAG8 expression shortened the hyperalgesia induced by the second acid injection. TDAG8 activation may regulate cAMP signalling to participate in initiating inflammatory hyperalgesia and establishing hyperalgesic priming.

## Results

### TDAG8 expression and function is knocked down by shTDAG8

**Figure 1:**
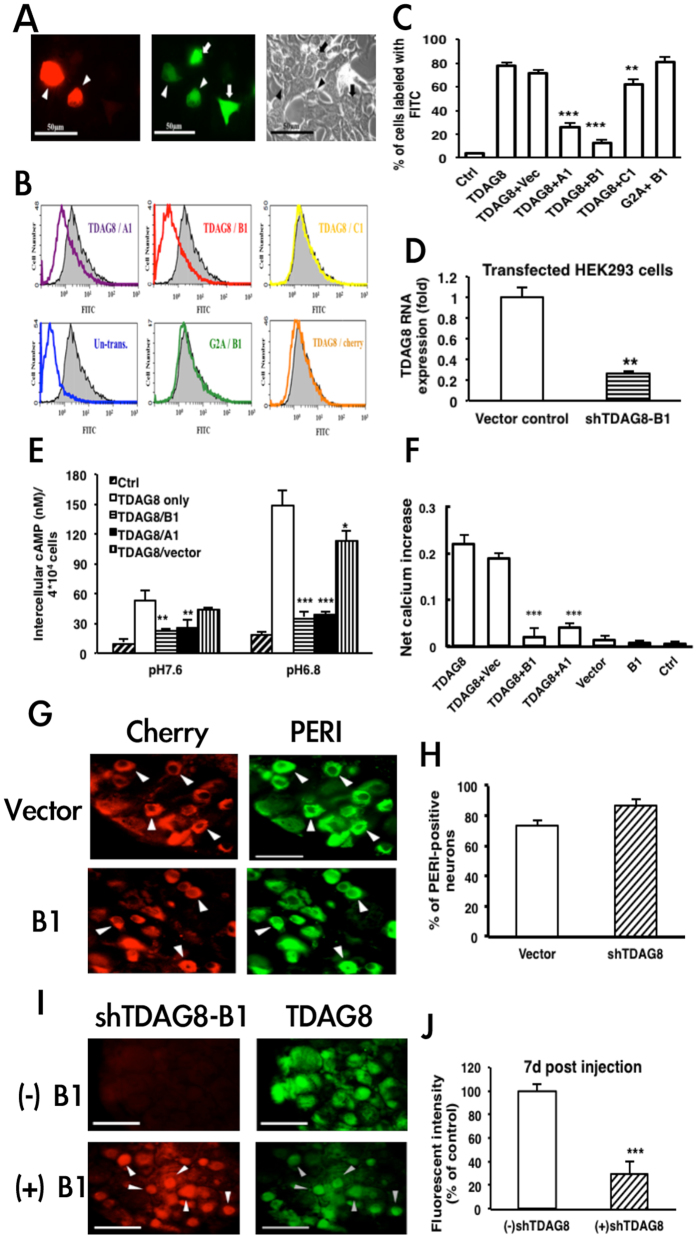
Knockdown efficiency of TDAG8-shRNA *in vitro* and *in vivo*. (**A–F**) HEK293T cells were left untransfected; transfected with TDAG8-EGFP; co-transfected with TDAG8-EGFP and shRNA-cherry vector, shTDAG8-A1, B1, or C1; or co-transfected with G2A-pEGFP and shTDAG8-B1 for 16 hr. (**A**) Images of cells co-expressing TDAG8 (green fluorescence) and shTDAG8-B1 (red fluorescence). Shows phase-contrast image. (**B**) Analysis of flow cytometry. The Y axis is the number of cells expressing TDAG8-EGFP. The X axis represents green fluorescence intensity (TDAG8 expression). The fluorescence intensity of untransfected cells (blue line) was <1 arbitrary unit, defined as the background level (ctrl). The grey area (black line) represents cells expressing TDAG8 alone. (**C**) The proportion of cells expressing TDAG8 from (**B**). **P < 0.01, ***p < 0.001 compared to TDAG8 alone by one-way ANOVA. (**D**) qRT-PCR of TDAG8 mRNA level in cells co-transfected with TDAG8-EGFP and cherry vector or shTDAG8-B1. **P < 0.01 by one-way ANOVA. (**E,F**) Transfected cells were stimulated with pH 7.6 and 6.8 buffer, followed by (**E**) cAMP measurement, or stimulated with pH 5.5, followed by (**F**) Ca^2+^ recording. *p < 0.05, **P < 0.01, ***p < 0.001 compared to TDAG8 alone by one-way ANOVA. (**G–J**) shTDAG8-B1/CG or Cherry vector/CG was intraplantarly injected into mice and expressed for 7 days. Lumbar 4-6 DRG ipsilateral to injected paws were removed and sectioned, then immunostained with anti-peripherin (PERI) or anti-TDAG8 antibody. (**G**) Cell images at 7 days after shTDAG8-B1 or vector injection. Red fluorescence indicates shTDAG8-B1 or vector expression and green fluorescence PERI-positive neurons. Arrowheads are neurons expressing both shTDAG8-B1 and PERI or vector and PERI. Scale bars are 50 μm. (**H**) Proportion of red fluorescent neurons to total PERI-positive neurons. (**I**) Cell images at 7 days after shTDAG8-B1 injection. Red fluorescence indicates shTDAG8-B1 expression and green fluorescence TDAG8-positive neurons. Arrowheads are neurons expressing both shTDAG8-B1 and TDAG8. Scale bars are 50 μm. Green fluorescence intensity is represented as the percentage of control (vector control) in (**J**). ***P < 0.001 by one-way ANOVA.

To understand whether TDAG8 is involved in inflammatory hyperalgesia, we first subcloned TDAG8-shRNA plasmids (shTDAG8-A1, -B1, -C1) into pLKO.1-Cherry vector (Table 1) and tested their knockdown efficiency on co-transfection with TDAG8-EGF in HEK293T cells. shTDAG8 expression reduced the fluorescence intensity of TDAG8-EGFP in co-transfected cells (Fig. 1A). The fluorescence intensity of TDAG8-EGFP–expressing cells was reduced with shTDAG8-A1 or -B1 co-transfection (Fig. 1B). The number of TDAG8–expressing cells was greatly reduced 66.8% and 83.5% with shTDAG8-A1 and -B1, respectively, and slightly reduced (16.8%) with shTDAG8-C1, with no decrease with the cherry vector control (0%) (Fig. 1C). We chose shTDAG8-B1 for the remaining experiments because of its greater knockdown efficiency.

shTDAG8-B1 did not decrease the number of G2A-expressing cells (0% reduction, Fig. 1C), which suggests that the inhibitory effect of shTDAG8-B1 was specific to the TDAG8 gene. The mRNA expression of TDAG8 was reduced 80% in cells co-transfected with shTDAG8-B1 (Fig. 1D). With decreased pH, cAMP accumulation was increased in TDAG8-transfected cells and peaked at pH 6.8 (Fig. 1E). In the presence of shTDAG8-B1, cAMP accumulation was inhibited in TDAG8-transfected cells (Fig. 1E), so shTDAG8-B1 may inhibit TDAG8-mediated cAMP accumulation. Similar results were obtained with shTDAG8-A1. At low pH (pH 5.5), TDAG8 mediated a Ca^2+^ signal, and Ca^2+^ signals were specifically inhibited by shTDAG8-A1 and -B1 (Fig. 1F).

We delivered shTDAG8-B1 or cherry vector plasmids intraplantarly into mouse DRG by using the CG delivery system. At 7 days after shTDAG8-B1 or vector injection, 87 ± 4% and 73 ± 4% of peripherin-positive neurons expressed shTDAG8-B1 and cherry vector, respectively (Fig. 1G,H). To test 7-day TDAG8 protein expression, we co-stained DRG from shTDAG8-B1 or uninjected mice with anti-TDAG8 antibody. Fluorescence intensity of TDAG8 was reduced 70% in shTDAG8-B1–injected DRG neurons (Fig. 1I,J).

### Inhibition of TDAG8 expression delays the onset of hyperalgesia and shortens inflammatory hyperalgesia

**Figure 2:**
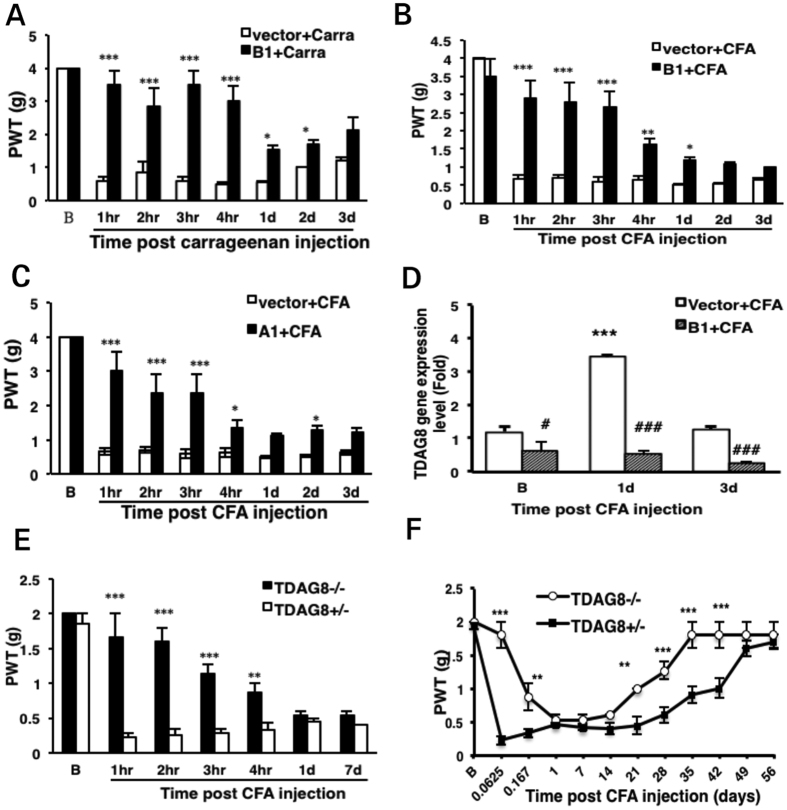
Suppressed TDAG8 expression or deletion of TDAG8 inhibits mechanical hyperalgesia induced by complete Freund’s adjuvant (CFA) or carrageenan. (**A–D**) Mice underwent intraplantar injection with shTDAG8-A1 or B1/CG (1:7.5) and at 7 d, were injected with carrageenan (20 mg/ml, carra, A) or CFA (50%, **B–D**). (**A–C**) Paw withdrawal threshold (PWT) was measured before (t = B) and after injection. Data are mean ± SEM of n ≥ 6 mice per group. *p < 0.05, **p < 0.01, ***p < 0.001 for shTDAG8 vs vector by two-way ANOVA. (**D**) qRT-PCR of TDAG8 mRNA level in lumbar 4–6 DRG collected before (**B**) or at 1, and 3 days after CFA injection at 7 days with shTDAG8-B1 or vector injection. ***p < 0.001 for B vs 1d, ^###^p < 0.001 for shTDAG8 vs vector by two-way ANOVA. (**E,F**) TDAG8^+/−^ or TDAG8^−/−^ mice underwent intraplantar injection with CFA (50%), followed by mechanical tests. Data are mean ± SEM of n ≥ 3 mice per group. **p < 0.01, ***p < 0.001 for TDAG8^+/−^ vs TDAG8^−/−^ by two-way ANOVA.

We then examined whether TDAG8 is involved in mechanical hyperalgesia induced by inflammation. shTDAG8-B1 was delivered into mice before carrageenan injection 7 days later. Knocked-down TDAG8 expression effectively inhibited hyperalgesia in the first 4 hr and the inhibitory effect was gradually reversed on day 1 after carrageenan injection (Fig. 2A). Similar results were found with CFA injection (Fig. 2B). The decreased inhibitory effect after day 1 was not due to the shRNA clone because similar results were found with shTDAG8-A1 (Fig. 2C).

To understand the decreased inhibitory effect of TDAG8 on hyperalgesia, we analysed the mRNA expression of TDAG8 mRNA before and after CFA injection in shTDAG8-B1– or vector-injected mice. TDAG8 expression was reduced 50% on day 7 after shTDAG8-B1 injection (Fig. 2D, 0 d CFA injection). At day 1 after CFA injection, TDAG8 gene expression was increased approximately three-fold as compared to 0 day. However, shTDAG8-B1 effectively suppressed TDAG8 gene expression on day 1 and day 3 (Fig. 2D). Therefore, decreased inhibition in hyperalgesia was not due to TDAG8 knockdown efficiency. More likely, TDAG8 plays a major role in hyperalgesia initiation rather than maintenance.

Similar results were found in TDAG8-knockout mice (Fig. 2E), showing delayed onset of mechanical hyperalgesia. Interestingly, deletion of TDAG8 gene also shortened hyperalgesia induced by CFA. Inflammatory hyperalgesia returned to the baseline level at week 5 in TDAG8^−/−^ mice but at week 7 in TDAG8^+/−^ mice (Fig. 2F). Accordingly, these data suggest that TDAG8 may be involved in the initiation of hyperalgesia and establishment of hyperalgesic priming.

### Inhibition of TDAG8 expression blocks cAMP accumulation and Ca^2+^ signals

**Figure 3:**
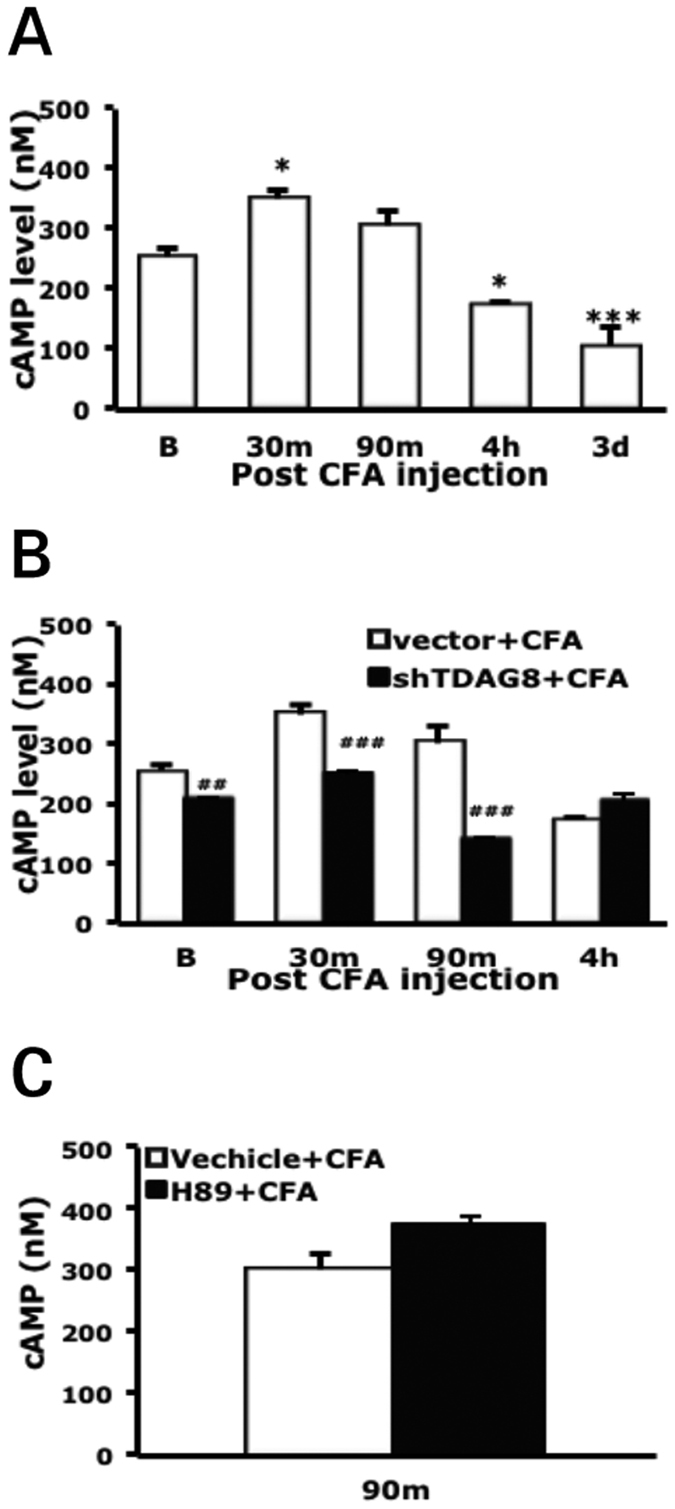
Intracellular cAMP accumulation is reduced by suppressed TDAG8. cAMP measurement in DRG ipsilateral or contralateral to the mouse paw that underwent intraplantar injection with (**A,B**) vector/CG or shTDAG8-B1/CG for 7 days, followed by 50% CFA injection or (**C**) saline or protein kinase A inhibitor H89, followed by 50% CFA injection. *p < 0.05, ***p < 0.001 for B vs 30 min, 4 hr, 3 d by one-way ANOVA. ^###^p < 0.001 for shTDAG8-B1 vs vector by two-way ANOVA.

TDAG8 participated in regulating the early phase of hyperalgesia induced by carrageenan or CFA in our study, which corresponds to PKA signalling regulation in early hyperalgesia^3^. Given that TDAG8 activation induces cAMP accumulation, leading to downstream PKA activation, TDAG8 likely regulates PKA to initiate the development of hyperalgesia. We then examined cAMP levels in DRG. After CFA injection, cAMP level was increased and peaked at 30 min, then decreased at 4 hr and 3 days (Fig. 3A), which suggests that cAMP signaling is essential in the initial phase (<4 hr). With shTDAG8-B1 treatment, cAMP level was kept at lower levels, with 20% lower before CFA injection, 30% at 30 min, and 54% at 90 min as compared to the vector control (Fig. 3B). The results suggest that knockdown of TDAG8 expression reduced cAMP accumulation. Because PKA is the downstream effector of cAMP, inhibition of PKA is not expected to affect cAMP accumulation. As expected, the PKA inhibitor H89 did not reduce cAMP level at 90 min (Fig. 3C). TDAG8 knockdown blocked cAMP accumulation in DRG but only in the initial phase (<4 hr). This result corresponds to behavioural data (Fig. 2B) and a previous study by Huang *et al*.^3^ (2015), which suggests that TDAG8 mediates the cAMP-PKA pathway to regulate the initial phase of inflammatory hyperalgesia.

**Figure 4:**
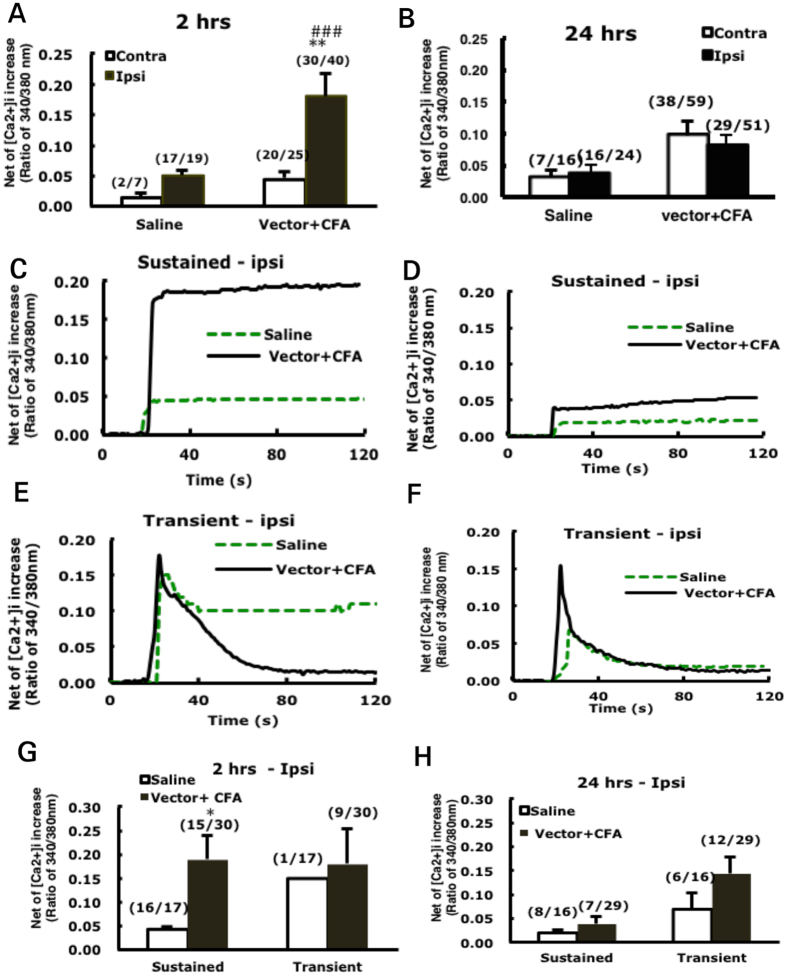
Intracellular Ca^2+^ levels ([Ca^2+^]_i_) increased at 2 hr after CFA injection. (**A,B**) L4-6 DRG were collected at 2 or 24 hr after mice underwent intraplantar injection with 50% CFA, and neurons were isolated and cultured for 12 hr. [Ca^2+^]_i_ signals were recorded in cultured neurons with exposure to pH 6.8 buffer. Time course of [Ca^2+^]_i_ signals after the addition of pH 6.8 in ipsilateral DRG neurons is shown in (**C–F**). Data points represent peak [Ca^2+^]_i_ signals (approximately 20 sec after the addition of pH buffer) (**A,B,G,H**). Data are mean ± SEM. The fraction in parentheses indicates the responding neurons to total collected cells. (**A**) ^###^p < 0.001 between CFA-ipsi and -contra. **p < 0.01 between CFA- and saline-ipsi; (**G**) *p < 0.05 between CFA- and saline-sustained.

To determine whether TDAG8 knockdown also affected acid-induced Ca^2+^ signals, DRG were isolated at 2 or 24 hr after CFA injection and cultured. At 2 hr after injection, pH 6.8 acidic buffer significantly increased [Ca^2+^]_i_ level in ipsilateral DRG neurons (Fig. 4A) but not contralateral DRG neurons. Two types of [Ca^2+^]_i_ signals were identified: one sustained and the other transient. At 2 hr, CFA injection largely increased sustained but not transient signals as compared to the saline control (Fig. 4C,E,G). Although the transient signals were not elevated, the proportion of neurons generating transient signals was increased (from 6% to 30%). CFA injection in the early phase may increase the proportion of neurons with transient signals and elevate sustained signals. At 24 hr, acid-evoked [Ca^2+^]_i_ signals were reduced in ipsilateral DRG neurons (Fig. 4B), mainly because of reduced sustained signals compared to at 2 hr after CFA injection (Fig. 4D,F,H). These results suggest that the response of DRG neurons to acid is increased in the initial phase after CFA injection, which could be relevant in the development of hyperalgesia.

**Figure 5:**
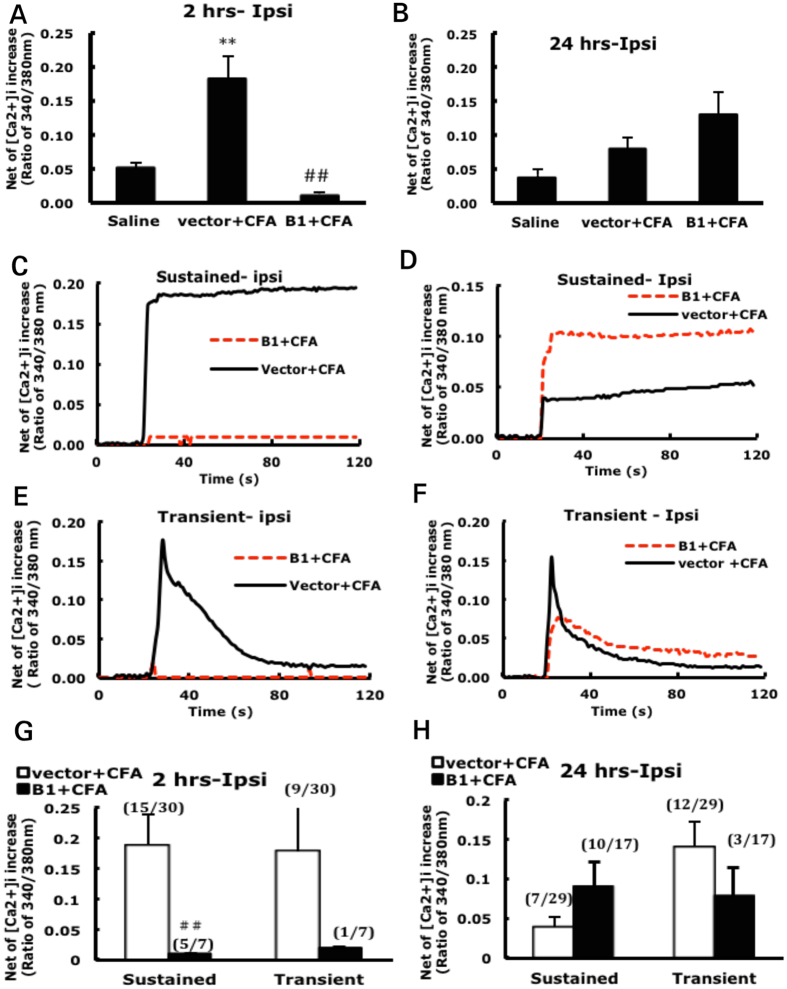
[Ca^2+^]_i_ level inhibited by suppressed TDAG8 expression. (**A,B**) L4-6 ipsilateral DRG were collected at 2 or 24 hr after 50% CFA injection in shTDAG8-B1 or vector-injected mice, and neurons were isolated and cultured for 12 hr. [Ca^2+^]_i_ signals were recorded in cultured neurons with exposure to pH 6.8 buffer. Time course of [Ca^2+^]_i_ signals after the addition of pH 6.8 shown in (**C–F**). Data points represent peak [Ca^2+^]i signals (approximately 20 sec after the addition of pH buffer) (**A,B,G,H**). Data are mean ± SEM. The fraction in the parentheses indicates the responding neurons to total collected cells. (**A**) ^##^p < 0.01 between CFA and B1+CFA. **p < 0.01 between CFA- and saline-ipsi; (**G**) ^##^p < 0.01 between CFA- and B1+CFA-ipsi.

We then examined shTDAG8-B1 effects. At 2 hr after administration of shTDAG8-B1, CFA-elevated [Ca^2+^]_i_ signals were markedly reduced in ipsilateral DRG neurons (Fig. 5A). Both sustained and transient signals were inhibited (Fig. 5C,E,G). TDAG8 knockdown did not reduce the [Ca^2+^]_i_ signals in ipsilateral DRG at 24 hr (Fig. 5B,D,F,H). In contrast, [Ca^2+^]_i_ signals in the ipsilateral side were slightly elevated (Fig. 5B). Thus, TDAG8 knockdown inhibited CFA-elevated [Ca^2+^]_i_ signals only at 2 hr after CFA injection. These results are also consistent with cAMP and hehavioral data, which implies that TDAG8 mediates cAMP-PKA and calcium signals to regulate the initial phase of inflammatory hyperalgesia.

### Inhibition of TDAG8 shortens the mechanical hyperalgesia induced by dual acid injection

**Figure 6:**
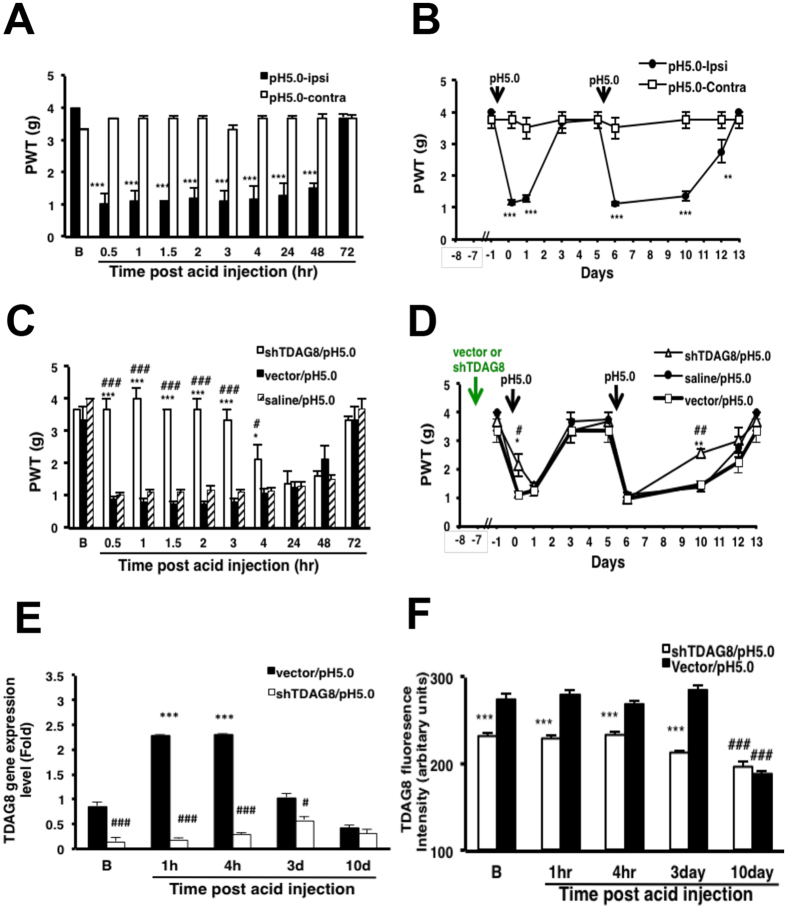
Suppressed TDAG8 expression shortens hyperalgesia induced by a second acid injection. (**A–D**) PWT measured in mice before (t = B) and with intraplantar pre-injection with 12.5 μg shTDAG8-B1/CG or vector/CG for 7 days, then injection with acid (pH 5.0), then with acid (pH 5.0) again 5 days later. Data are mean ± SEM of n ≥ 6 mice per group. **P < 0.01 and ***p < 0.001 for ipsi vs contra; *p < 0.05, **P < 0.01, and ***p < 0.001 for shTDAG8 vs vector by two-way ANOVA. (**E**) qRT-PCR of mRNA level of TDAG8 in lumbar 4-6 DRG collected from mice at B, 1, 4 hr, 3d or 10d after acid injection at 7 days after shTDAG8 or vector injection. ***P < 0.001 for B vs 1 hr or 4 hr; ^#^p < 0.05, ^###^P < 0.001 for shTDAG8 vs vector by two-way ANOVA. (**F**) Protein level of TDAG8 in lumabr 5 DRG collected from mice at B, 1, 4 hr, or 3d after acid injection at 7 days after shTDAG8 or vector injection. ***P < 0.001 for shTDAG8 vs vector; ^###^P < 0.001 for B vs 10d by two-way ANOVA.

We have found that mice lacking TDAG8 gene show shortened hyperalgesia, which suggests that TDAG8 may be involved in hyperalgesic priming^4^,^5^. To further confirm whether TDAG8 is required for hyperalgesic priming, we used dual acid injection to examine the priming effect. The first acid injection (pH 5.0) immediately induced acute unilateral hyperalgesia, which peaked at 30 min and returned to baseline on day 3 (Fig. 6A). At day 5 after the first injection, acid was injected again and hyperalgesia lasted longer, for 8 days (Fig. 6B). Hyperalgesia was not found on contralateral sides.

TDAG8-shRNA plasmids were delivered to mice, then at 7 days, mice were injected with acid buffer (pH 5.0) and underwent mechanical testing. With 7-day shTDAG8-B1 expression, the inhibitory effect of shTDAG8-B1 lasted for 4 hr (Fig. 6C). After the second acid injection, the hyperalgesia was only maintained for 5 days (8 days for controls) (Fig. 6D). Therefore, administration of shTDAG8-B1 inhibited the initial phase of the first hyperalgesia and also shortened hyperalgesia induced by the second acid injection.

The short inhibitory effect of shTDAG8-B1 could not be due to incomplete suppression of TDAG8 gene or protein. We examined TDAG8 gene expression before (B) or at 1, and 4 hr, 3 and 10 days after acid injection. In vector control, TDAG8 gene level was increased two-fold with acid injection at 1 hr and 4 hr, but the level was returned to baseline at 3 days and slightly declined at 10 days. shTDAG8-B1 injection decreased TDAG8 gene expression (85% reduction) at 0 hr, and a low level of TDAG8 was maintained for at least 10 days (Fig. 6E). We also examined protein levels in DRG using immunostaining and found that TDAG8 protein levels were unchanged in vector control from 1 hr to 3 days after acid injection, but slightly declined at 10 days. shTDAG8-B1 injection suppressed TDAG8 protein levels at a lower level (Fig. 6F).

**Figure 7:**
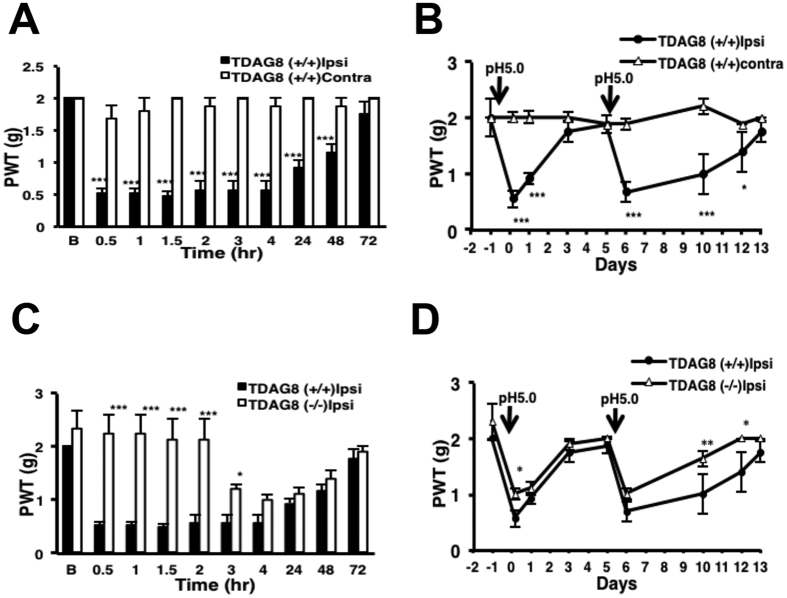
Deletion of TDAG8 gene shortens hyperalgesia induced by the second acid injection. (**A–D**) PWT measured in TDAG8^+/+^ or TDAG8^−/−^ mice before (t = B), then after injection with acid (pH 5.0), then acid (pH 5.0) again 5 days later. Data are mean ± SEM of n ≥ 6 mice per group. **P < 0.01 and ***p < 0.001 for Ipsi vs contra; *p < 0.05, **P < 0.01, and ***p < 0.001 for shTDAG8 vs vector by two-way ANOVA.

We used TDAG8^+/+^ and TDAG8^−/−^ mice to confirm the results. In TDAG8^+/+^ mice, the first acid injection induced hyperalgesia at 30 min, which returned to baseline on day 3; the second acid injection caused 8-day-long hyperalgesia (Fig. 7A,B). In TDAG8^−/−^ mice, the hyperalgesia induced by the first acid injection was inhibited in the first 3 hr (Fig. 7C). The hyperalgesia caused by the second acid injection was shortened to 5 d (Fig. 7D), which suggests that deletion of TDAG8 delayed the first hyperalgesia and shortened the second hyperalgesia.

**Figure 8:**
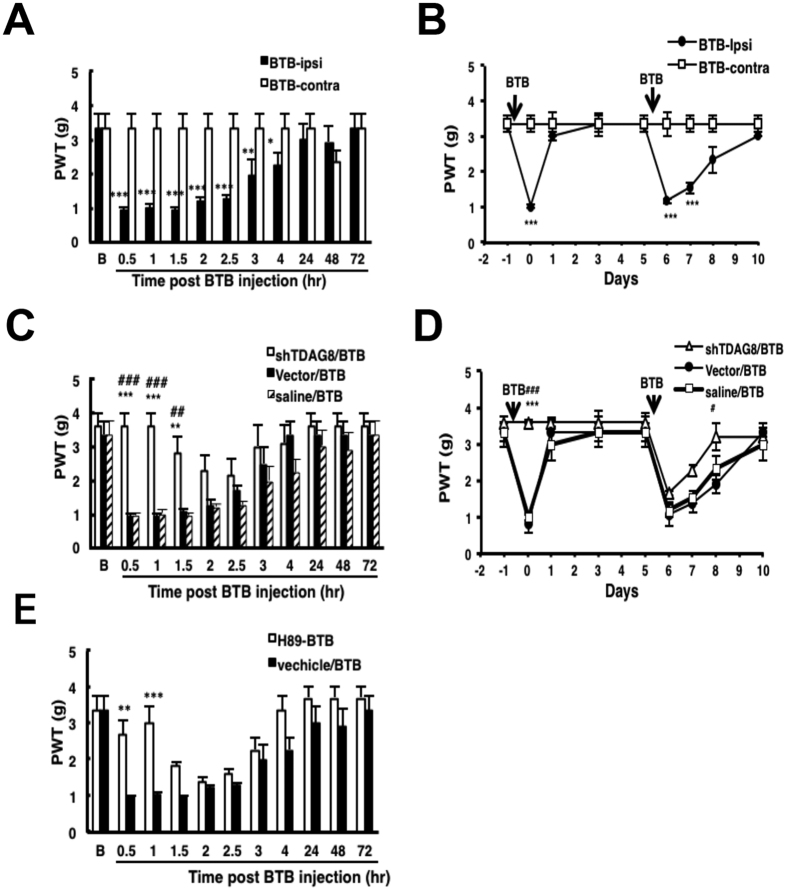
Dual administration of TDAG8 agonists induces a longer hyperalgesia in the second injection. (**A,B**) PWT measured in mice before (t = B), then injection with TDAG8 agonist (BTB09089, BTB, 100 μM), then with BTB09089 again 5 days later. (**C,D**) PWT measured in mice before (t = B) and with and without intraplantar pre-injection with 12.5 μg shTDAG8-B1/CG or vector for 7 days, then injection with BTB, then BTB again 5 days later. (**E**) PKA inhibitor, H89 (50 μM) was pre-injected, followed by BTB injection. Data are mean ± SEM of n ≥ 6 mice per group. *p < 0.05, **P < 0.01 and ***p < 0.001 for Ipsi vs contra; ^#^p < 0.05, ^##^P < 0.01, and ^###^p < 0.001 for shTDAG8 vs vector; **P < 0.01, and ***p < 0.001 for shTDAG8 vs saline*; **P < 0.01, and ***p < 0.001 for H89 vs vechicle control by two-way ANOVA.

We then injected the TDAG8 agonist BTB09089^14^ to confirm the function of TDAG8 in hyperalgesic priming. Administration of TDAG8 agonist induced mechanical hyperalgesia at 30 min, which lasted for 4 hr (Fig. 8A). At 5 day after the first injection, the TDAG8 agonist was injected again and mechanical hyperalgesia was maintained for at least 3 days (Fig. 8B). Knockdown of TDAG8 expression reversed the BTB09089-induced first hyperalgesia (1.5 hrs inhibition) and shortened the second hyperalgesia (2 days) (Fig. 8C,D), which further confirmed that TDAG8 is involved in initiating hyperalgesia and establishing hyperalgesic priming. Similar results were found by inhibiting PKA activity with H89 (Fig. 8E), which suggests that TDAG8 initiates hyperalgesia and mediates hyperalgesic priming via PKA signalling.

## Discussion

We have demonstrated that deletion of TDAG8 or long-term knockdown of TDAG8 gene expression by shRNA specifically targeting TDAG8 delayed the onset of acid or inflammation-induced hyperalgesia and also shortened hyperalgesia. In a dual acid-injection model, deletion of TDAG8 and TDAG8 knockdown shortened the second-acid–induced hyperpalgesia. Administration of a TDAG8 agonist initiated mechanical hyperalgesia and induced hyperalgesic priming. TDAG8 agonist-induced hyperalgesia and hyperalgesic priming was inhibited by TDAG8 knockdown and use of a PKA inhibitor. Accordingly, TDAG8 is required for initiating hyperalgesia and establishing hyperalgesic priming.

We selected two shRNA clones (shTDAG8-A1 and -B1) targeted to TDAG8 and stably expressed them *in vitro* and *in vivo*. Both clones (A1 and B1) inhibited the mRNA and protein expression of TDAG8 and inhibited TDAG8 function in cultured cells or neurons (Fig. 1). Although shTDAG8 continuously suppressed TDAG8 gene expression, it completely inhibited CFA- or carrageenan-induced hyperalgesia only in the early phase (<4 hr). Because TDAG8 gene expression was continuously suppressed by shTDAG8 (>50% inhibition), the failure to inhibit hyperalgesia in the later phase (after 4 hr) could not be attributed to incomplete suppression of TDAG8 expression. TDAG8-knockout mice also showed less 4-hr inhibition, which confirms that TDAG8 could be essential in initiating rather than maintaining hyperalgesia. A previous study by Huang^3^ *et al*. found a kinase-dependent transition in acid- or CFA-induced hyperalgesia. PKA regulates the first 4 hr, whereas PKCε controls the prolonged phase after 4 hr. Huang^3^ *et al*. suggested that the acidosis signal is responsible for the kinase-dependent transition via proton-sensing G-protein-coupled receptors. We found immediately increased level cAMP (a upstream regulator of PKA) at 30 min after CFA injection, which decreased after 4 hr (Fig. 3). The time of high levels of cAMP corresponds to the time of PKA action. Deletion or suppression of TDAG8 gene expression reduced cAMP accumulation and inhibited hyperalgesia in the first 4 hr, which corresponds to the action time of PKA signaling. Administration of a TDAG8 agonist induced short-term hyperalgesia, which could be inhibited by the PKA inhibitor H89 (Fig. 8E). These results suggested that TDAG8 mediates the initiation of hyperalgesia via cAMP-PKA signaling.

TDAG8 is involved in the initiation of hyperalgesia and also affects the establishment of hyperalgesic priming. In the CFA model, TDAG8 deletion shortened the inflammatory hyperalgesia. The dual acid-injection model revealed that inhibition of TDAG8 gene expression shortened the hyperalgesia induced by the second acid injection. Administration of an TDAG8 agonist twice 5 days apart caused a longer hyperalgesia after the second injection. These results confirmed that TDAG8 is involved in hyperalgesic priming. Thus, TDAG8-PKA signaling is important to establish the chronic pain state, although it may not be required for maintaining the chronic state. TDAG8-mediated signaling may regulate [Ca^2+^]_i_ signals to establish hyperlagesic priming. TDAG8 regulates acid-induced [Ca^2+^]_i_ *in vitro*^9^ (Fig. 1F) and *in vivo*^11^ (Figs 4 and 5). Analysis of neuronal [Ca^2+^]_i_ signals revealed two patterns of [Ca^2+^]_i_ signals (sustained and transient). At 2 hr after CFA injection, sustained but not transient [Ca^2+^]_i_ signals were increased, although the number of neurons with transient signals was increased. At 24 hr after CFA injection, sustained [Ca^2+^]_i_ signals returned to the basal level. Such [Ca^2+^]_i_ signals were suppressed by TDAG8 knockdown at 2 hr but not 24 hr. In contrast, TDAG8 knockdown increased sustained currents of [Ca^2+^]_i_. Accordingly, TDAG8 activation increased sustained currents of calcium signals in neurons to initiate hyperalgesia (the first 4 hr), then inhibited sustained signals to extend hyperalgesia. With suppressed TDAG8 expression, the initiation of hyperalgesia was inhibited, and hyperalgesia was shortened. However, how TDAG8 regulates calcium signals remains unclear, but we recently found that TDAG8 could regulate TRPV1 to increase [Ca^2+^]_i_ *in vitro*^16^. TDAG8 may regulate other channels to control [Ca^2+^]_i_. Alternatively, TDAG8 can induce cAMP accumulation, so cAMP probably activates the Epac pathway to increase [Ca^2+^]_i_.

Consistent with previous study^11^, we found increased peripheral TDAG8 expression in DRG at 1 day after CFA injection (Fig. 2D). However, TDAG8 mediates only the initial phase of hyperalgesia. At 1 day after injection, hyperalgesia was independent of TDAG8. Given that TDAG8 is expressed in neurons and also immune cells and microglia^10^,^13^,^14^,^15^, moderate TDAG8 expression in neurons may be sufficient to initiate hyperalgesia. At 1 day after CFA injection, the increased expression of TDAG8 in DRG could be due to an increase in glial cells rather than neurons. Thus, increased TDAG8 expression may be more important in the inflammatory process and less important in neuron activity. Although glial cells also have some roles in maintenance of hyperalgesia^17^, TDAG8 activation in glial or immune cells aims to inhibit production of pro-inflammatory cytokines^13^,^14^,^15^. Thus, deletion of TDAG8 in glial cells may further promote inflammation, thereby leading to enhanced hyperalgesia. It could explain in part why inhibition of TDAG8 expression at 1 day (>4 hr) did not reduce hyperalgesia. Analysis of DRG neuronal [Ca^2+^]_i_ signals revealed that suppressed TDAG8 expression inhibited both sustained and transient signals at 2 hr but not 24 hr. These results support a role for TDAG8 in neurons mainly in the initiation rather than maintenance of hyperalgesia.

Therefore, in neurons, TDAG8 could receive acidosis signals, then induce downstream second-messenger pathways to regulate channels (such as TRPV1), to lead to pain and hyperalgesia. In contrast, TDAG8 in macrophages or microglia may respond to acidosis signals to decrease the production of cytokines, thereby attenuating hyperalgesia. Given that neurons play a dominant role in the initiation of hyperalgesia, knocked-down TDAG8 expression or deletion of TDAG8 in neurons completely inhibited CFA-induced hyperalgesia in the beginning of hyperalgesia. Because the maintenance of hyperalgesia requires some other cells than neurons, suppression of TDAG8 expression in neurons may not have effective influence on the maintenance. However, knocked-down or deleted TDAG8 shortened chronic hyperalgesia, implying that establishment of hyperalgesic priming requires TDAG8. Having said that, the non-cell-autonomous effect of TDAG8 cannot be excluded. We have examined other proton-sensing receptors in DRG from KD or KO mice. Only TRPV1 gene expression was down-regulated before CFA or acid stimulation. We has previously found TDAG8 can regulate TRPV1^11^. Thus, TRPV1 may have contribution to the non-cell-autonomous effect of TDAG8.

## Conclusions

This is the first study to demonstrate that TDAG8 function is essential for inflammatory hyperalgesia and hyperalgesic priming. We have demonstrated that knocked-down TDAG8 expression in the periphery or deletion of TDAG8 delayed the onset of inflammatory hyperalgesia and shortened hyperalgesia in mice. A dual acid-injection model provided solid evidence that TDAG8 mediates the acidosis signal to establish hyperalgesic priming. The dual TDAG8 agonist model further confirmed that TDAG8 is involved in establishing hyperalgesic priming. Knocked-down TDAG8 inhibited neuronal calcium signals and cAMP accumulation, which suggests that TDAG8 mediates acidosis signals to initiate hyperalgesia and establish hyperalgesia priming through cAMP or calcium signals.

## Materials and Methods

### Agents and constructs

CFA, carrageenan, 2-(N-morpholino) ethanesulfonic acid (MES), and 4-(2-hydroxyethyl)-1-piperazineethanesulfonic acid (HEPES) were from Sigma. H89 dihydrochloride (N-[2-[[3-(4-Bromophenyl)-2-propenyl]amino]ethyl]-5-isoquinolinesulfonamide dihydrochloride) was from Tocris Bioscience. TDAG8 agonist BTB09089 was from Maybridge (North Cornwall, UK). Microsphere of cationized gelatin (CG) was kindly prepared and provided by Dr. Yasuhiko Tabata^18^ (Kyoto University, Japan). For animal experiments, all drugs or peptides were diluted into saline before injection.

TDAG8-shRNA plasmids (shRNA clones TRCN0000027417 [A01], TRCN0000027436 [B01], TRCN0000027459 [C01]) were from the Taiwan National RNAi Core Facility, Academia Sinica, Taiwan. TDAG8-shRNA plasmids A01, B01, and C01 (shTDAG8-A01, -B01, -C01) were subcloned into pLKO.1-Cherry vector (re-named shTDAG8-A1, B1, C1). The target sequence for TDAG8 is in Table 1. TDAG8 and TDAG8-shRNA plasmids were cloned into the vector pIRES-hrGFP-2a (pIRES-GFP) and pLKO.1-puro cherry, respectively, for transfection experiments.

### Animal experiments and tissue collection

ICR mice (8–12 weeks old) were purchased from BioLASCO Taiwan (Taipei), bred and cared for in accordance with the Guide for the Use of Laboratory Animals (National Academy Press, Washington DC). Mice were housed 3–4 per cage under a 12-h light/dark cycle (lights on at 7:00 am) with food and water *ad libitum* in a temperature and humidity controlled environment at the National Central University. Animal experimental procedures were approved by the local animal use committee (IACUC, National Central University, Taiwan). Behavioural testing was performed between 9:00 am and 5:00 pm. Effort was made to minimize the number of animals used and their suffering. TDAG8^+/+^, TDAG8^+/−^, TDAG8^−/−^ mice were generated and bred as described^13^. The primer sequences for genotyping were for TDAG8^−/−^, forward, 5′-cgaactctagctggcttttatccaataat-3′, and reverse, 5′-cttgtgtcatgcacaaagtagatgtcc-3′.

Mice underwent intraplantar injection with 25 μl saline, carrageenan (20 mg/ml), or CFA (50% in saline) before cell or animal behaviour experiments. In a dual acid-injection model, mice were intraplantarly injected with 25 μl acid (pH 5.0) twice, 5 days apart. For gene, tissue or cell experiments, mice were placed in the euthanasia chamber and sacrificed by introducing 100% carbon dioxide with a fill rate of 20–30%/min. Mice were unconscious usually within 2 to 3 minutes. After sacrifice, lumbar 4-6 (L4-6) DRG ipsilateral and contralateral to injected paws were removed at the indicated times for RNA extraction, tissue extraction, or primary cell culture.

In some experiments, mice were pre-injected with 25 or 12.5 μg shTDAG8/CG polyplex (1:7.5) or vector/CG before acid, carrageenan, or CFA injection 7 days later. The preparation of shTDAG8/CG was as described^18^,^19^. Briefly, plasmid DNA (shTDAG8) and CG were each heated to 65 °C for 10 min and separately diluted into 5% glucose. CG was added into plasmid DNA at a weight ratio of 7.5:1 and vortexed for 30 sec before injection in mice.

Behavioral experiments were mainly done using male mice, while gene expression and cellular experiments were using male and female mice. Female mice were tested and have shown similar behaviors as male mice, so they were used in gene and cellular experiments.

### Cell culture and transfection

Human embryonic kidney 293 cells (HEK293T, obtained from the Bioresource Collection and Research Center of Food Industry Research and Development Institute, Taiwan) were cultured and maintained in DMEM (Invitrogen) supplemented with 10% fetal bovine serum (Invitrogen) and antibiotics as described^11^. For Ca^2+^ imaging experiments, HEK293T cells were seeded at 4 × 10^5^ on 24-mm poly-D-lysine-coated coverslips, transfected with 1.5 μg of the plasmid pIRES-GFP-TDAG8, pIRES-GFP-G2A, pLKO-cherry-TDAG8shRNA, pIRES-GFP, or pLKO-cherry, then underwent Ca^2+^ imaging. For cAMP assay, HEK293T cells were seeded at 1.6 × 10^5^ per well (70–80% confluence) in 12-well plates, transfected with 1.5 μg of the plasmid pIRES-GFP-TDAG8, pLKO-cherry-TDAG8shRNA, pIRES-GFP, or pLKO-cherry, then underwent cAMP assay. In some experiments, cells were co-transfected with 1.5 μg of both pIRES-GFP-TDAG8 and pLKO-cherry-TDAG8shRNA or pIRES-GFP-TDAG8 and pLKO-cherry at a ratio of 1:1.

Primary DRG were cultured as described^11^. Briefly, ICR mice were pre-injected with or without shTDAG8-B1, then injected with 50% CFA or saline. At 2 or 24 hr post-CFA injection, mouse lumbar 4-6 (L4-6) DRG were collected, treated with 0.125% collagenase IA (Sigma) and digested with 0.25% trypsin. Cells were re-suspended, collected and centrifuged at 1224×g for 5 min. The cell pellet was suspended, mixed and cultured for 12 hr before Ca^2+^ imaging.

### Mechanical test

The pain behaviour test was described previously^20^. Male ICR mice with or without treatment were tested for withdrawal thresholds to mechanical stimuli (von Frey filaments, Touch-Test, North Coast Medical, Morgan Hill, CA) applied to the hindpaw. Mice (n ≥ 6 per group) were pre-trained for 1 to 2 hr each day for 2 days before the test. Von Frey fibers were applied 5 times at 5-sec intervals to the plantar surface of each hindpaw at various times after injections. The paw withdrawal threshold (PWT) was determined when paw withdrawal was observed in more than 3 of 5 applications.

### Immunohistochemistry and flow cytometry

Mice were intraplantarly injected with shTDAG8/CG, then 4 or 7 days later, L4-6 DRGs were obtained and immediately placed into freezing solution. Serial sections 12-μm thick were cut by use of a cryostat (Leica microsystem 3510S, Bensheim, Germany). Sections were washed with 1× phosphate buffered saline (PBS), then stained with primary antibodies anti-peripherin (1:500, Sigma) or anti-TDAG8 (1:100, custom by Genesis, Taiwan), then FITC-conjugated goat-anti-rabbit-IgG antibody (1:250, Sigma). All antibodies were diluted in 1× PBS containing 1% bovine serum albumin. All incubations were carried out at 4 °C overnight. The specimens were examined under a Lecia DMI3000B fluorescence microscope (Germany) and digitized images were captured by use of MetaVue software.

For flow cytometry, transfected cells were trypsinized and suspended in DMEM. After centrifugation at 1000× g for 5 min, cell pellets were re-suspended in PBS, centrifuged at 1000 g for 5 min, re-suspended in 70% ethanol/PBS, then underwent flow cytometry (Beckman Coulter). The FITC channel was used to analyze the fluorescence intensity for TDAG8-EGFP and red fluorescence channel for shTDAG8.

### cAMP assay

Transfected HEK293T cells were pre-incubated for 15 min with serum-free DMEM containing 30 μM of the phosphodiesterase inhibitor RO201724 (Sigma), then stimulated with indicated pH buffers containing 30 μM RO201724 for 30 min at 37 °C. After stimulation, cells were lysed in ethanol. The lysates were dried, and cAMP level in dried lysates was quantified by use of the cAMP immunoassay kit (Assay Designs, MI) according to the manufacturer’s protocol. For DRG tissue, L4-6 DRG were flash-frozen in liquid nitrogen and ground to a fine powder under liquid nitrogen. After liquid nitrogen evaporated, 0.1 M HCl (about 300 μl, 10-fold volume frozen tissue) was added into the ground powder. After centrifugation at 600× g for 10 min to remove debris, the supernatant was directly used in the assay or stored frozen for later analysis.

### Imaging of intracellular Ca^2+^ level ([Ca^2+^]_i_)

[Ca^2+^]_i_ imaging was performed as described^11^. Briefly, cells grown on coverslips were pre-incubated at 37 °C with 2.5 μM Fura-2 acetoxymethyl ester (Fura-2-AM, Molecular Probes) for 40 min in HEPES/MES buffer (125 mM NaCl, 1 mM KCl, 5 mM CaCl_2_, 1 mM MgCl_2_, 8 mM glucose, 10 mM HEPES and 15 mM MES, pH 7.4). After being washed, cells were supplemented with 300 μl HEPES/MES buffer (pH 7.6), then stimulated with pH 6.8 or 5.5 HEPES/MES buffer (600 μl). The pH-evoked Ca^2+^ transients were recorded by use of a Ca^2+^ imaging system equipped with a Leica DMI3000B fluorescence microscope and analyzed by use of MetaFluor software.

### RNA preparation and quantitative RT-PCR

DRG RNA extraction was performed as described^10^. Each DRG pool contained at least 9 to 12 DRG from 3 to 4 mice. RNA was extracted by using the RNeasy kit (Qiagen, Valencia, CA). The gene primers (100 nM), derived cDNA, and master mix (SYBR green I and AmpliTaq Gold DNA polymerase [Applied Biosystems, Foster City, CA]) were mixed for PCR reactions and product detection by use of ABI Prism 7300. For each assay, preparations were run in triplicate or quadruplicate. The thermal cycling conditions were 95 °C for 10 min, followed by 40 cycles of 95 °C for 15 s, and 60 °C for 1 min. The threshold cycle (Ct) values of both the targets and internal reference (mGAPDH) were measured from the same samples, and target gene expression relative to that of mGAPDH was calculated by the comparative Ct method.

The primer sequences were for TDAG8, forward, 5′-atagtcagcgtcccagccaac-3′, and reverse, 5′-cgcttcctttgcacaaggtg-3′; and mGAPDH, forward, 5′-ggagccaaacgggtcatcatctc-3′, and reverse, 5′-gaggggccatccacagtcttct-3′, as an internal control.

### Statistical analysis

All data are presented as mean ± SEM. One-way or two-way ANOVA with post-hoc Bonferroni correction was used to compare results from multiple groups. P < 0.05 was considered statistically significant.

## Additional Information

**How to cite this article**: Dai, S.-P. *et al*. TDAG8 involved in initiating inflammatory hyperalgesia and establishing hyperalgesic priming in mice. *Sci. Rep.* **7**, 41415; doi: 10.1038/srep41415 (2017).

**Publisher's note:** Springer Nature remains neutral with regard to jurisdictional claims in published maps and institutional affiliations.

## Figures and Tables

**Table 1:** TDAG8-shRNA clone sequence.

Clone ID	Clone name	Vector	Target sequence	Oligo sequence
TRCN0000027417	shTDAG8-A1	pLKO.1-cherry	CCACAAGAGATGCTGTAGAAT	CCGGCCACAAGAGATGCTGTAGAATCTCGAGATTCTACAGCATCTCTTGTGGTTTTT
TRCN0000027436	shTDAG8-B1	pLKO.1-cherry	CCAGCCAACATCGGATCTTTA	CCGGCCAGCCAACATCGGATCTTTACTCGAGTAAAGATCCGATGTTGGCTGGTTTTT
TRCN0000027459	shTDAG8-C1	pLKO.1-cherry	CCTTGTGCAAAGGAAGCGTTT	CCGGCCTTGTGCAAAGGAAGCGTTTCTCGAGAAACGCTTCCTTTGCACAAGGTTTTT
